# Supplementary material for: Free-breathing phase-resolved functional lung (PREFUL) low-field magnetic resonance imaging (LF-MRI) of pulmonary dysfunction after surviving childhood cancer
Source: Commun Med (Lond). 2026 Jan 22;6:99. doi: 10.1038/s43856-025-01365-w (PMC12891585; doi:10.1038/s43856-025-01365-w)
Supplement: Supplementary file 2 — Description of Additional Supplementary files [file 43856_2025_1365_MOESM2_ESM.pdf]

Description of Additional Supplementary Files

File name: Supplementary Data 1

Description: Clinical characteristics and results of the MRI examinations of the study participants.

File name: Supplementary Data 2

Description: Results of the statistical analysis.
